# Supplementary material for: Development of long lifespan high-energy aqueous organic||iodine rechargeable batteries
Source: Nat Commun. 2022 Oct 30;13:6489. doi: 10.1038/s41467-022-34303-8 (PMC9618581; doi:10.1038/s41467-022-34303-8)
Supplement: Supplementary file 1 — Supplementary Information [file 41467_2022_34303_MOESM1_ESM.pdf]

SUPPLEMENTARY INFORMATION

Development of long lifespan high-energy aqueous organic||iodine rechargeable batteries

Zishuai Zhang[1,2,3], Yilong Zhu[4], Miao Yu[1*], Yan Jiao[4*] and Yan Huang [2,3,5*]

[1]State Key Laboratory of Urban Water Resource and Environment, School of Chemistry and Chemical Engineering, Harbin Institute of Technology, Harbin, 150001, China

[2]Sauvage Laboratory for Smart Materials, School of Materials Science and Engineering, Harbin Institute of Technology, Shenzhen, 518055, China

[3]Shenzhen Key Laboratory of Flexible Printed Electronics Technology, Harbin Institute of Technology, Shenzhen, 518055, China

[4]School of Chemical Engineering & Advanced Materials, The University of Adelaide, Adelaide, SA 5005, Australia

[5]State Key Laboratory of Advanced Welding and Joining, Harbin Institute of Technology, Harbin, 150001, China

* Corresponding author.

Correspondence to:

miaoyu_che@hit.edu.cn; yan.jiao@adelaide.edu.au; yanhuanglib@hit.edu.cn

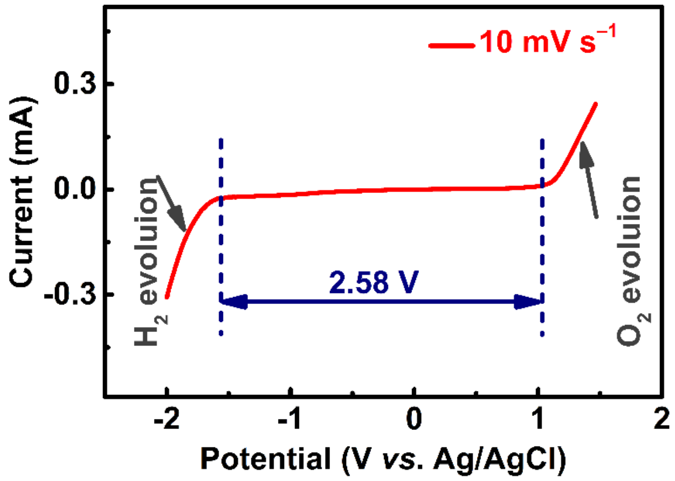

Supplementary Figure 1. LSV curve of the saturated KCl electrolyte. LSV curve of the saturated KCl electrolyte (10 mL) in a three-electrode glass cell system (working electrode: Ketjen Black (KB); counter electrode: platinum foil; reference electrode: standard Ag/AgCl) at 25±1 °C.

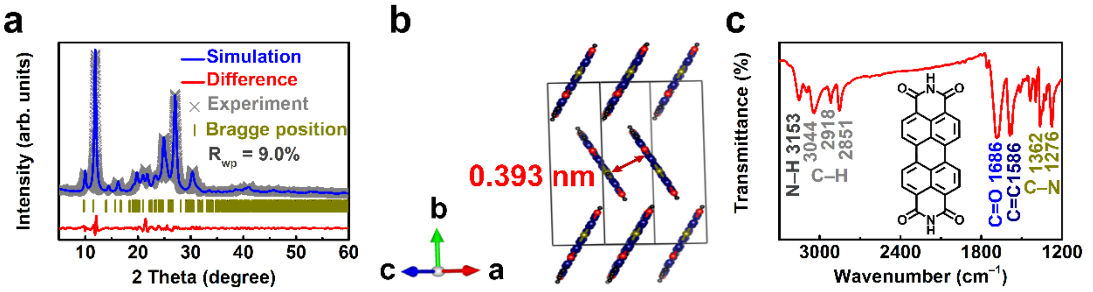

Supplementary Figure 2. XRD and FT-IR measurements for PTCDI powder. a, XRD pattern of PTCDI powder (grey crosses) and the Rietveld–refined XRD pattern (blue line). b, The structural stacking diagram of PTCDI. c, ATR–FTIR spectrum of PTCDI powder.

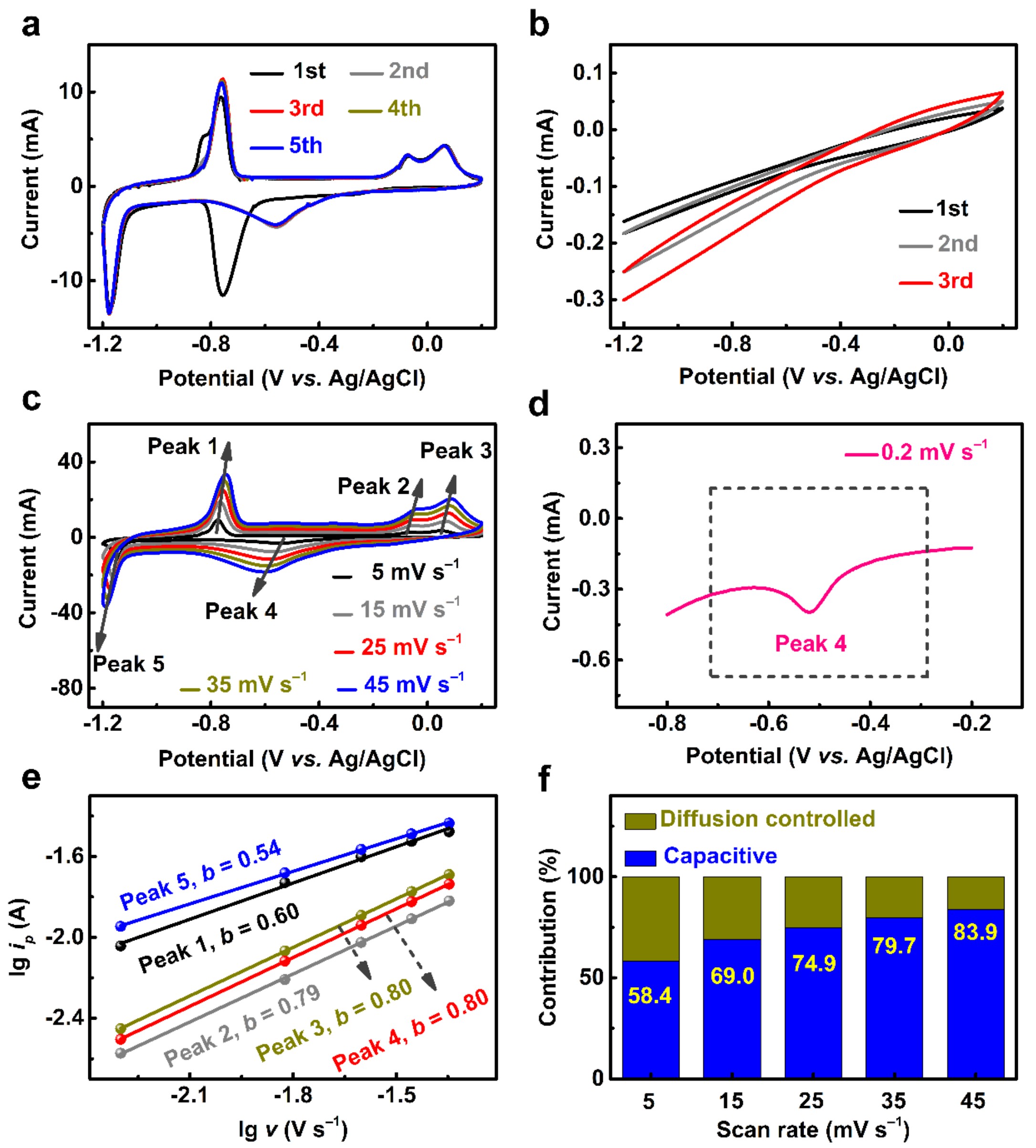

Supplementary Figure 3. Reaction kinetics of the PTCDI electrode in a saturated KCl aqueous electrolyte. a, CV curves of the PTCDI electrode in a three-electrode glass cell system (working electrode: PTCDI; counter electrode: platinum foil; reference electrode: standard Ag/AgCl; electrolyte: 10 mL saturated KCl solution) at 10 mV s^{-1} and 25±1 °C. b, CV curves of the PTCDI electrode through a three-electrode glass cell system (working electrode: PTCDI; counter electrode: platinum foil; reference electrode: standard Ag/AgCl) in a dilute HCl solution (pH ≈ 5, 10 mL) at 10 mV s^{-1} and 25±1 °C. c, CV curves of the PTCDI electrode in a three-electrode glass cell system (working electrode: PTCDI; counter electrode: platinum foil; reference electrode:

standard Ag/AgCl; electrolyte: 10 mL saturated KCl solution; 25±1 °C) at various scan rates. d, Peak 4 in the CV curve of the PTCDI electrode in a three-electrode glass cell system (working electrode: PTCDI; counter electrode: platinum foil; reference electrode: standard Ag/AgCl; electrolyte: 10 mL saturated KCl solution) at 0.2 mV s^{-1} and 25±1 °C. e, The corresponding lg i_p *vs*. lg v plots at different redox peaks. f, Contribution ratio of the capacitive- and diffusion-controlled process at different scan rates.

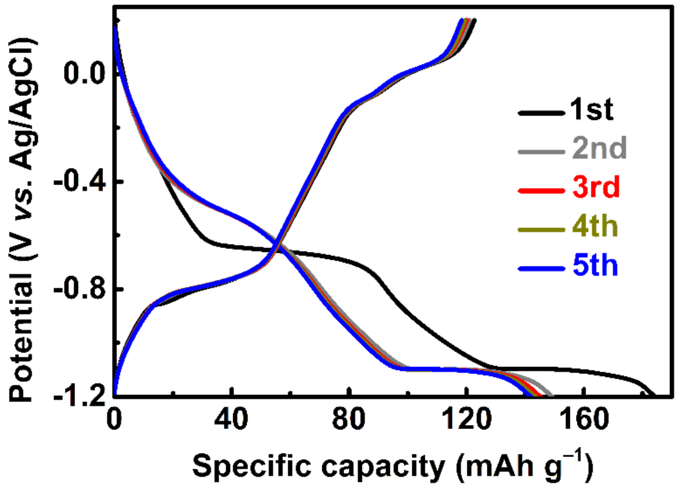

Supplementary Figure 4. Electrochemical performance of the PTCDI electrode. GCD curves of the PTCDI electrode in a three-electrode glass cell configuration (working electrode: PTCDI; counter electrode: platinum foil; reference electrode: standard Ag/AgCl; electrolyte: 10 mL saturated KCl solution) at 1.2 A g^{-1} and 25±1 °C.

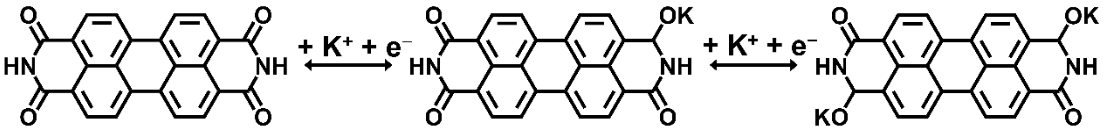

Supplementary Figure 5. The reaction mechanism of the PTCDI electrode. Schematics of the redox mechanism for the reversible K-ion storage reaction of the PTCDI electrode.

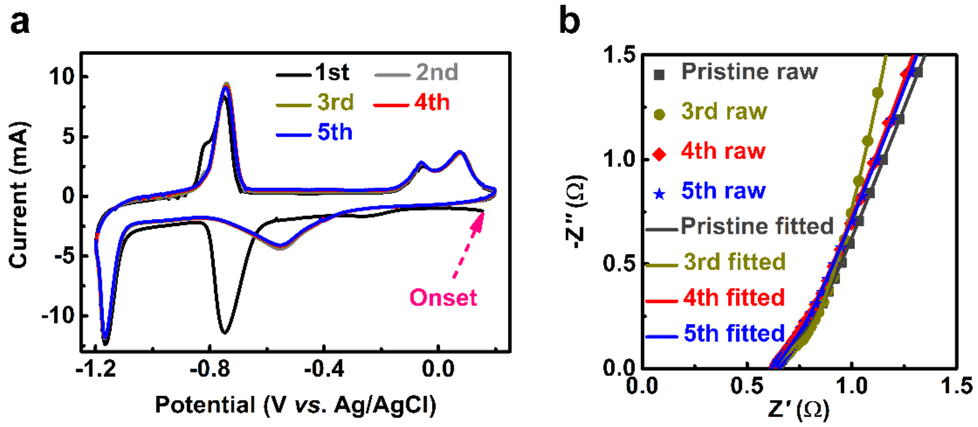

Supplementary Figure 6. Reaction kinetics of the PTCDI electrode in a saturated mixed KCl/I_2 aqueous electrolyte. a, CV curves of the PTCDI electrode in a three-electrode glass cell system (working electrode: PTCDI; counter electrode: platinum foil; reference electrode: standard Ag/AgCl; electrolyte: 10 mL saturated mixed KCl/I_2 aqueous electrolyte) at 10 mV s^{-1} and 25±1 °C. b, The corresponding EIS curves (the numerical values of the fitted EIS measurements are disclosed in supplementary table 2).

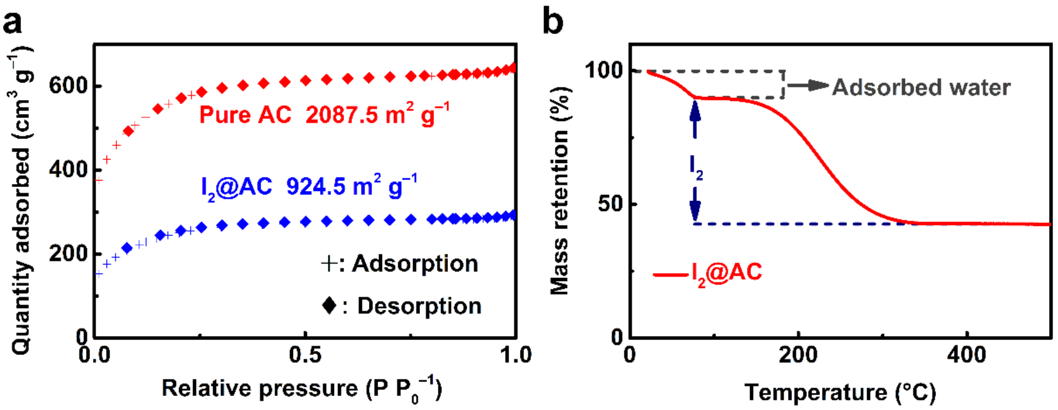

Supplementary Figure 7. BET and TGA measurements of AC and I_2@AC powder.

a, N_2 adsorption-desorption isotherms of pure active carbon (AC) and I_2@AC powder.

b, TGA curve of the synthesized I_2@AC powder.

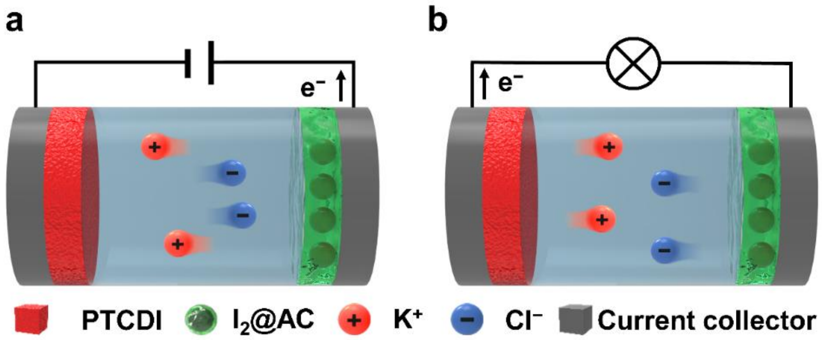

Supplementary Figure 8. Schematics of the reaction mechanism of the PTCDI||I_2 glass cell. a–b, Schematics of the reaction mechanism of the PTCDI||I_2 glass cell in a saturated KCl aqueous electrolyte during the charging and discharging processes, respectively.

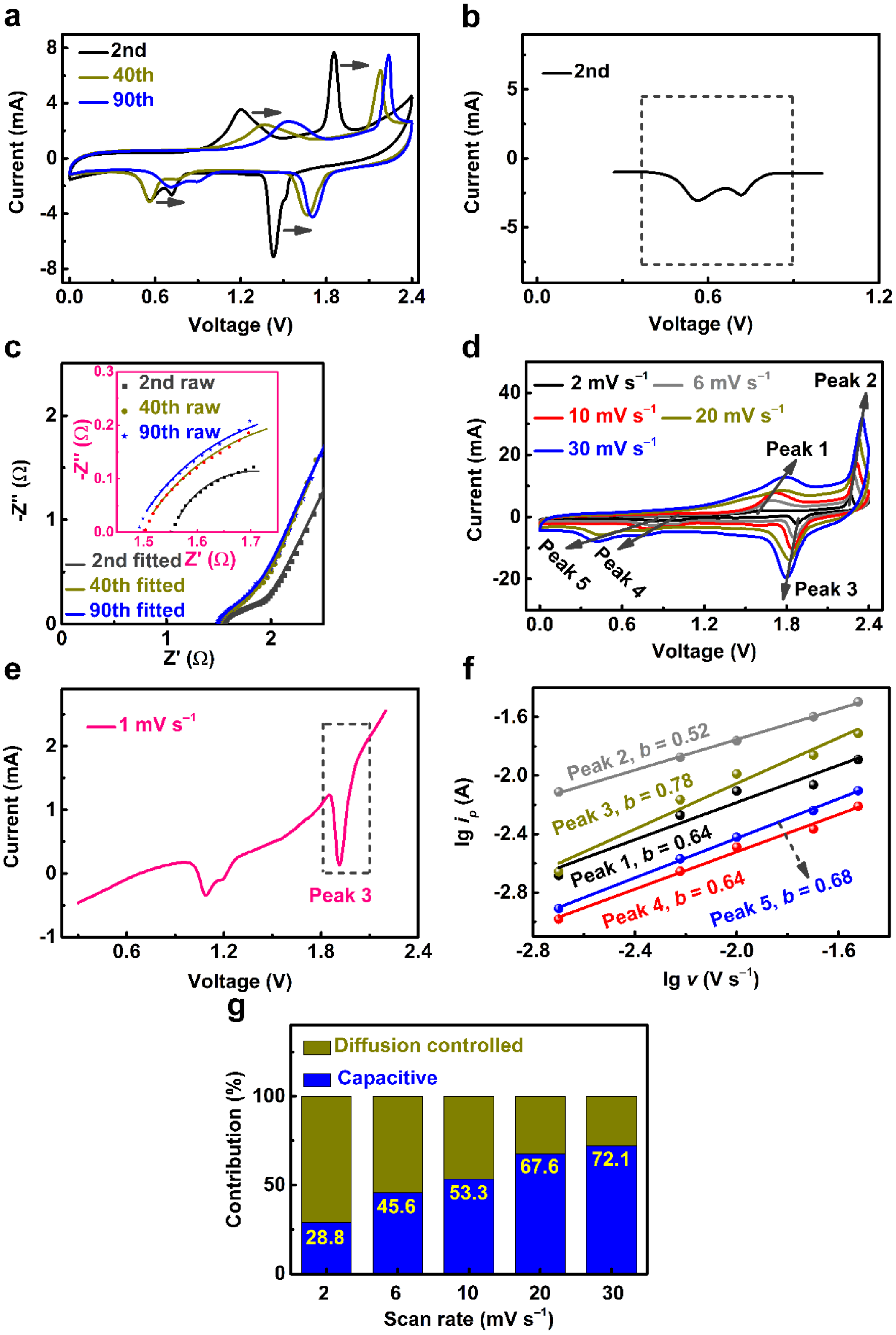

Supplementary Figure 9. Reaction kinetics of the PTCDI||I_2 glass cell in a saturated KCl aqueous electrolyte. a, CV curves of the PTCDI||I_2 in a two-electrode glass cell system (the positive electrode: the I_2 electrode; the negative electrode: the PTCDI electrode;

electrolyte: 10 mL saturated KCl aqueous solution) at 10 mV s^{-1} and 25±1 °C. b, The corresponding partial enlargement of the 2nd CV curve of the full cell. c, EIS curves (the numerical values of the fitted EIS measurements are disclosed in supplementary table 3) of the full cell after the 2nd, 40th and 90th CV tests at 10 mV s^{-1} and 25±1 °C . d, CV curves of the full cell in a two-electrode glass cell system (the positive electrode: the I_2 electrode; the negative electrode: the PTCDI electrode; electrolyte: 10 mL saturated KCl aqueous solution; 25±1 °C) at various scan rates. e, Peak 3 in the CV curve of the PTCDI||I_2 in a two-electrode glass cell system (the positive electrode: the I_2 electrode; the negative electrode: the PTCDI electrode; electrolyte: 10 mL saturated KCl aqueous solution) at 1 mV s^{-1} and 25±1 °C. f, The corresponding lg i_p *vs*. lg v plots at different redox peaks. g, Contribution ratio of the capacitance- and diffusion-controlled process at different scan rates.

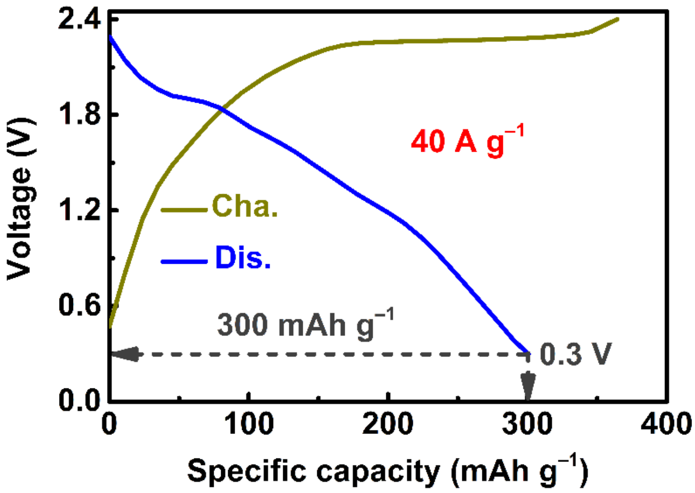

Supplementary Figure 10. GCD curve of the PTCDI||I_2 glass cell within 0.3−2.4 V.

The typical GCD curve of the PTCDI||I_2 in a two-electrode glass cell system (the positive electrode: the I_2 electrode; the negative electrode: the PTCDI electrode; electrolyte: 10 mL saturated KCl aqueous solution; 25±1 °C) within the voltage range of 0.3−2.4 V.

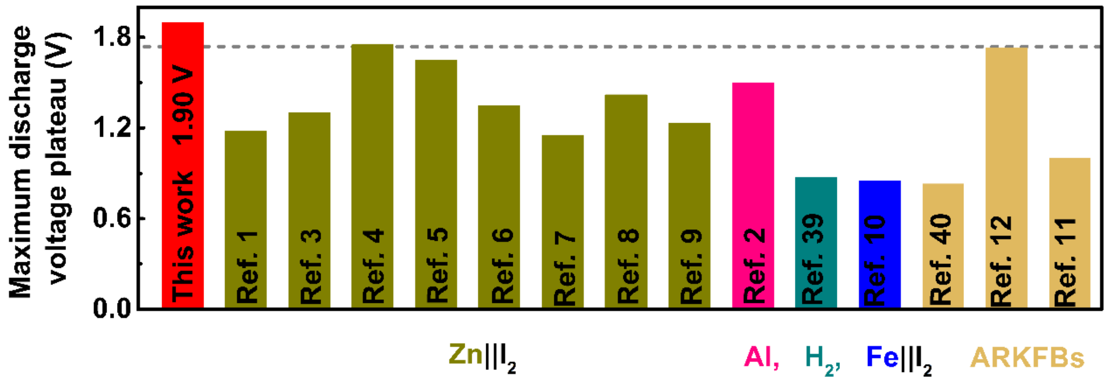

Supplementary Figure 11. The maximum discharge voltage plateau of the PTCDI||I_2 glass cell compared to reported works.

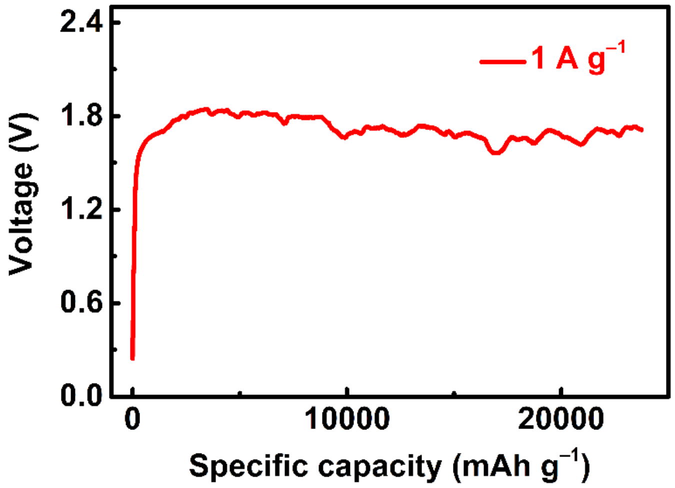

Supplementary Figure 12. GCD curve of the PTCDI||I_2 glass cell at 1 A g^{-1}. GCD curve collected from the first cycle of the PTCDI||I_2 in a two-electrode glass cell system (the positive electrode: the I_2 electrode; the negative electrode: the PTCDI electrode; electrolyte: 10 mL saturated KCl aqueous solution) at 1 A g^{-1} and 25±1 °C.

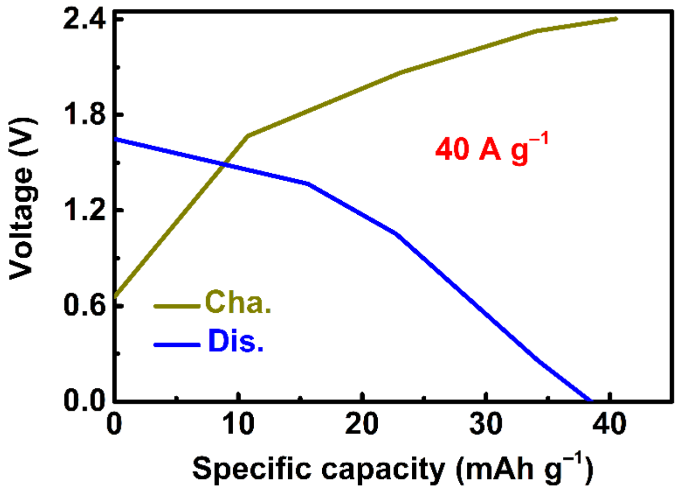

Supplementary Figure 13. Typical GCD curve of the PTCDI||AC glass cell. Typical GCD curve collected from the 1000th cycle of the PTCDI||AC in a two-electrode glass cell system (the positive electrode: the AC electrode; the negative electrode: the PTCDI electrode; electrolyte: 10 mL saturated KCl aqueous solution; 25±1 °C) (the specific capacity was calculated based on the mass loading of AC).

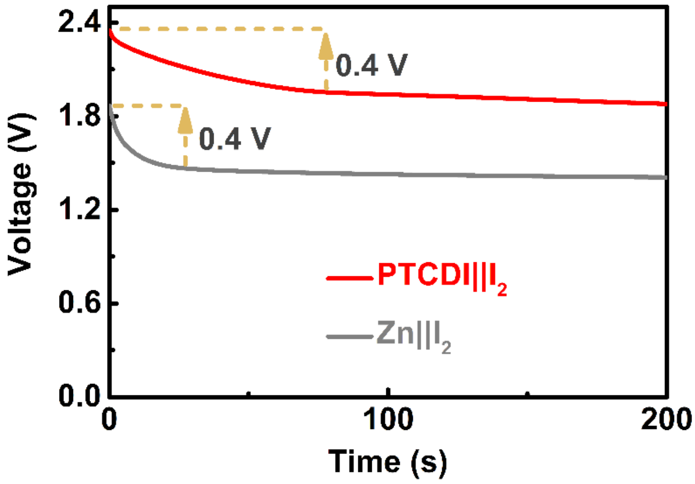

Supplementary Figure 14. Comparison of the self-discharge rate between the PTCDI||I_2 glass cell and Zn||I_2 glass cell. Comparison of the self-discharge rate between the PTCDI||I_2 glass cell (the positive electrode: the I_2 electrode; the negative electrode: the PTCDI electrode; electrolyte: 10 mL saturated KCl aqueous solution; 25±1 °C) and the Zn||I_2 glass cell (the positive electrode: the I_2 electrode; the negative electrode: the Zn electrode; electrolyte: 10 mL saturated KCl aqueous solution; 25±1 °C). The voltage profiles of both full cells were collected after the 10th cycle and the 11th charge process.

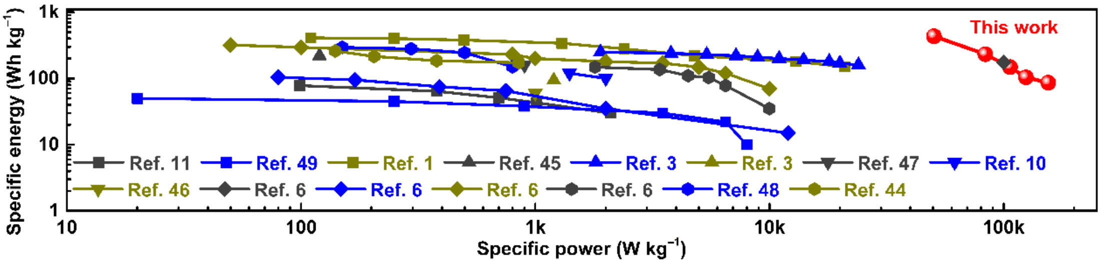

Supplementary Figure 15. The specific energy and power performances of the PTCDI||I_2 glass cell compared to the previously reported works. The specific energy and power performances of the PTCDI||I_2 glass cell compared to the previously reported aqueous I_2-cathode-based batteries and ARKFBs.

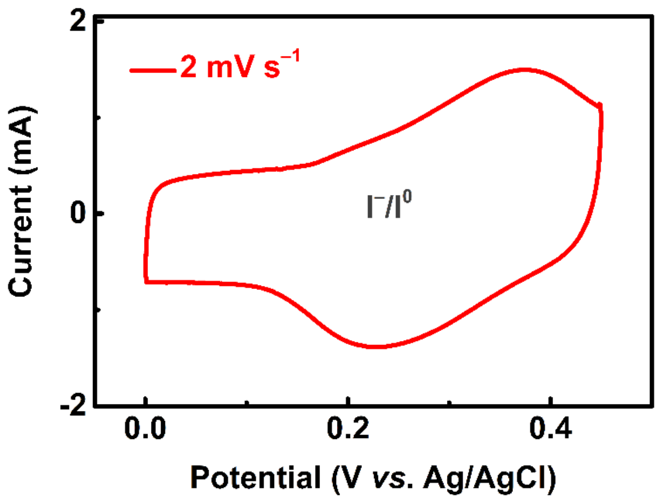

Supplementary Figure 16. CV curve of the I_2 electrode in a three-electrode glass cell system. CV curve of the I_2 electrode in a three-electrode glass cell system (working electrode: I_2@AC; counter electrode: platinum foil; reference electrode: standard Ag/AgCl; electrolyte: 10 mL saturated KCl solution) at 2 mV s^{-1} and 25±1 °C.

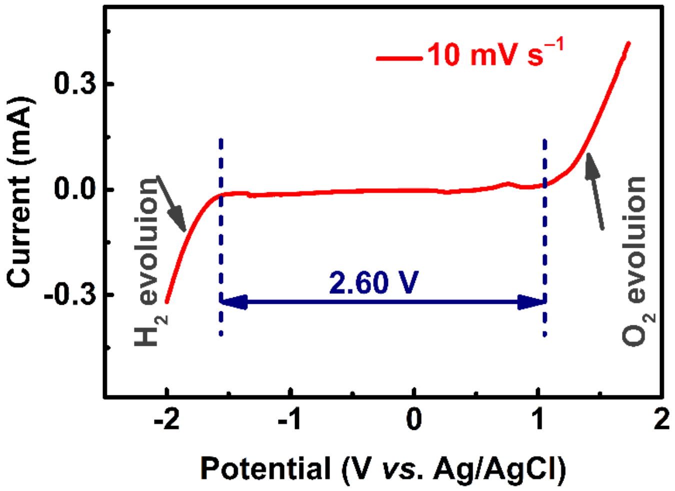

Supplementary Figure 17. LSV curve of the saturated mixed KCl/I_2 aqueous electrolyte. LSV curve of the saturated mixed KCl/I_2 aqueous electrolyte (10 mL) in a three-electrode glass cell system (working electrode: KB; counter electrode: platinum foil; reference electrode: standard Ag/AgCl) at 25±1 °C.

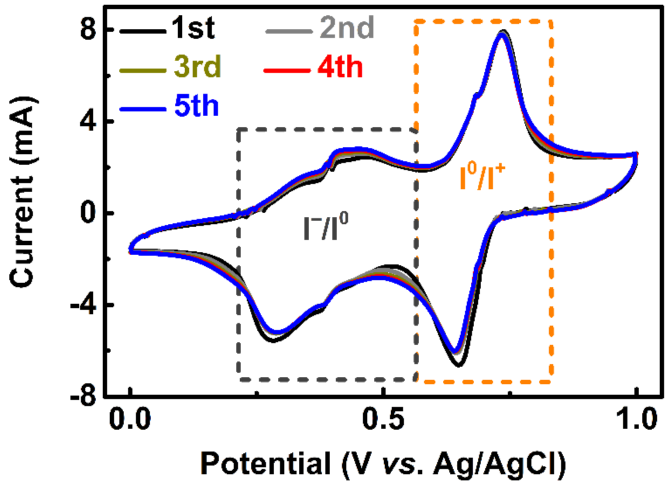

Supplementary Figure 18. CV curves of the CP electrode in a saturated mixed KCl/I_2 electrolyte. CV curves of the carbon paper (CP) electrode in a three-electrode glass cell system (working electrode: CP; counter electrode: platinum foil; reference electrode: standard Ag/AgCl; electrolyte: 10 mL saturated mixed KCl/I_2 solution) at 10 mV s^{-1} and 25±1 °C.

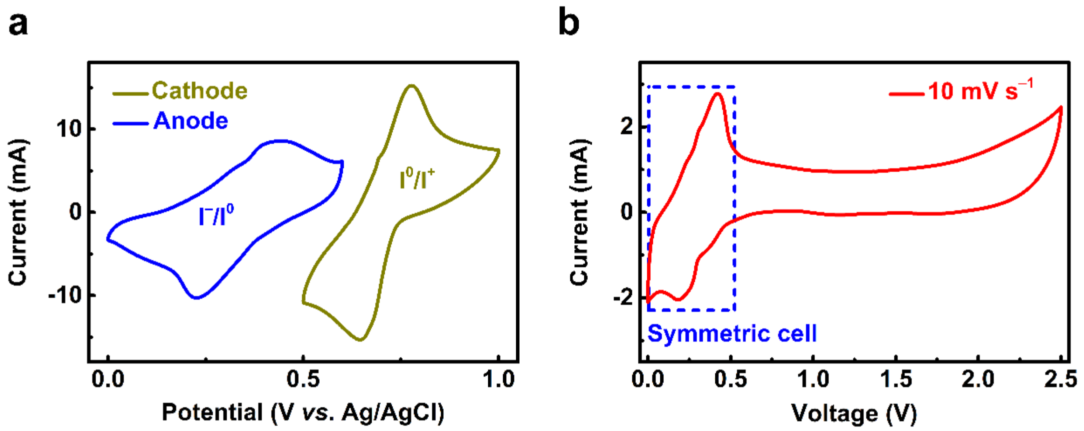

Supplementary Figure 19. Typical CV curve of the CP electrode and the CP||CP glass cell in a saturated mixed KCl/I_2 electrolyte. a, Typical CV curves of the carbon paper (CP) substrate in a three-electrode glass cell system (working electrode: CP; counter electrode: platinum foil; reference electrode: standard Ag/AgCl electrode; electrolyte: 10 mL saturated mixed KCl/I_2 solution) at 10 mV s^{-1} and 25±1 °C. b, Typical CV curve of the CP||CP in a two-electrode glass cell system (the positive electrode: CP; the negative electrode: CP; electrolyte: 10 mL saturated mixed KCl/I_2 solution) at 10 mV s^{-1} and 25±1 °C.

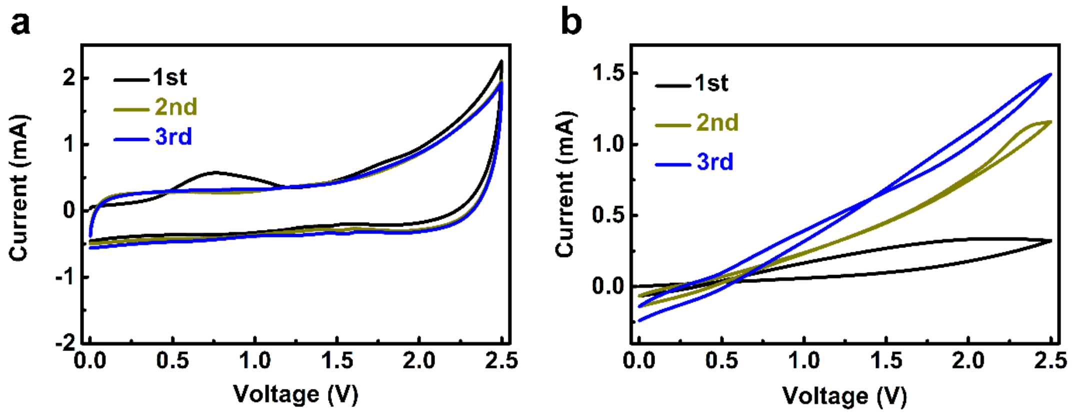

Supplementary Figure 20. CV curves of the CP||CP glass cell in a saturated KCl electrolyte and in a saturated I_2 electrolyte. a, CV curves of the CP||CP in a two-electrode glass cell system (the positive electrode: CP; the negative electrode: CP; electrolyte: 10 mL saturated KCl solution) at 10 mV s^{-1} and 25±1 °C. b, CV curves of the CP||CP in a two-electrode glass cell system (the positive electrode: CP; the negative electrode: CP; electrolyte: 10 mL saturated I_2 solution) at 10 mV s^{-1} and 25±1 °C.

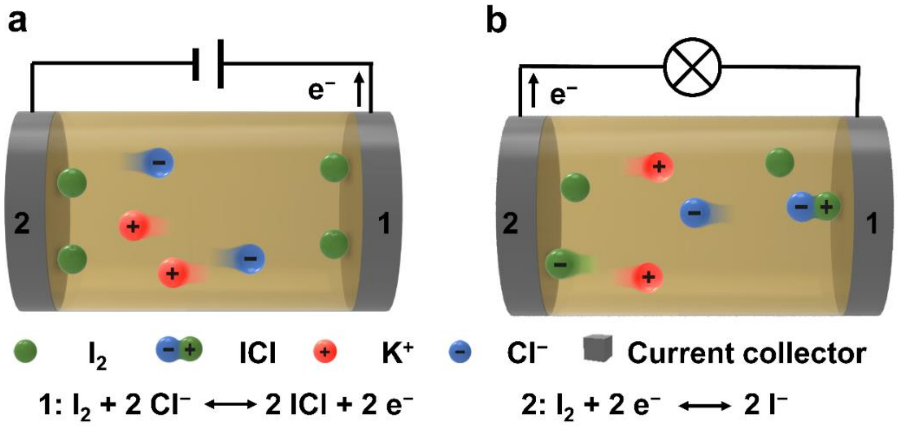

Supplementary Figure 21. Schematics of the CP||CP glass cell in a saturated mixed KCl/I_2 aqueous electrolyte. a–b, Schematics of the CP||CP glass cell (corresponding to the $I^-/I^0||I^0/I^+$ redox system) in a saturated mixed KCl/I_2 aqueous electrolyte during the charging and discharging processes, respectively.

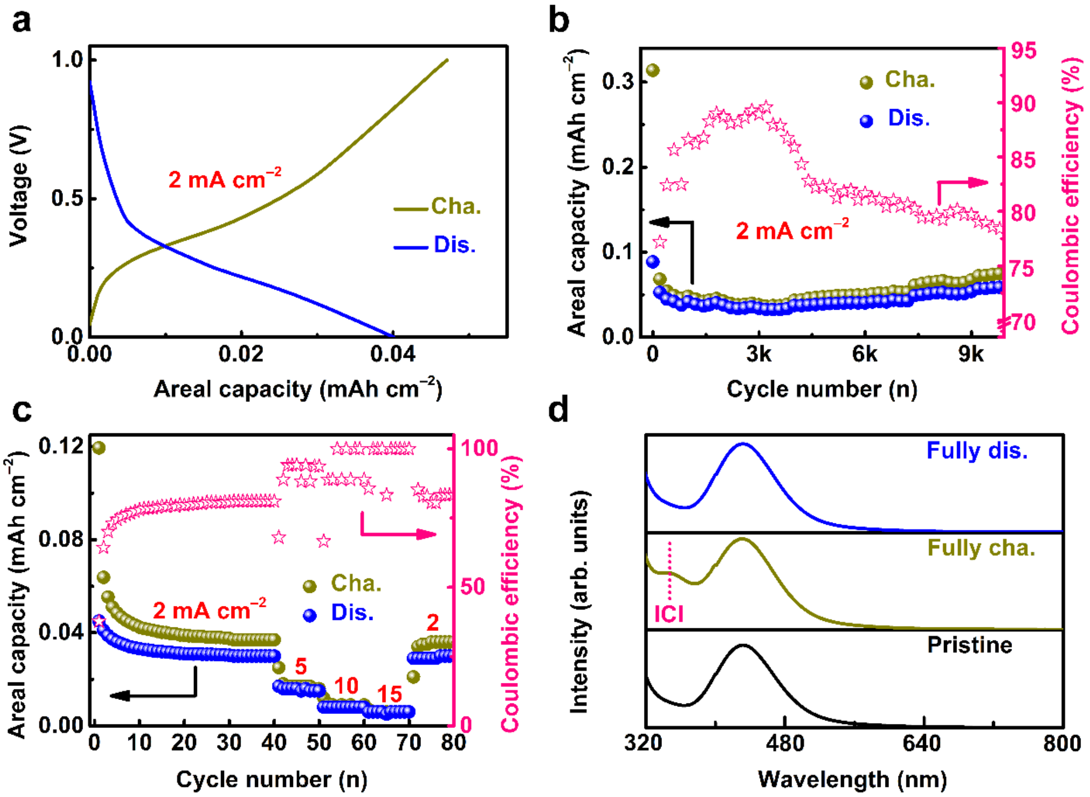

Supplementary Figure 22. Electrochemical performance of the CP||CP glass cell in a saturated mixed KCl/I_2 aqueous electrolyte. a–c, GCD curve collected from the 1000th cycle, cycling performance and rate capability of the CP||CP glass cell (corresponding to the $I^-/I^0||I^0/I^+$ redox system) in a saturated mixed KCl/I_2 aqueous electrolyte at 25±1 °C. d, Ex situ UV−*vis* spectra of the utilized electrolyte (the saturated mixed KCl/I_2 aqueous electrolyte used in the glass cell) recorded in the pristine (uncycled), fully charged and fully discharged states.

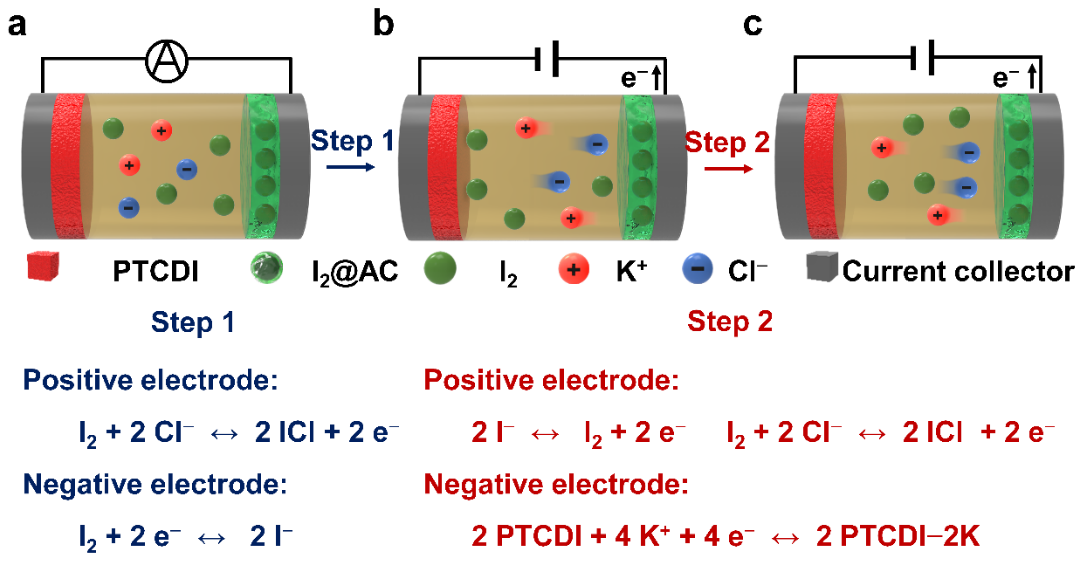

Supplementary Figure 23. Schematic illustration of the PTCDI||I_2 cascade glass cell in a saturated mixed KCl/I_2 aqueous electrolyte. a–c, Schematic illustration of the PTCDI||I_2 cascade glass cell in a saturated mixed KCl/I_2 aqueous electrolyte in the pristine (uncycled) state, the first charge step and the second charge step, respectively.

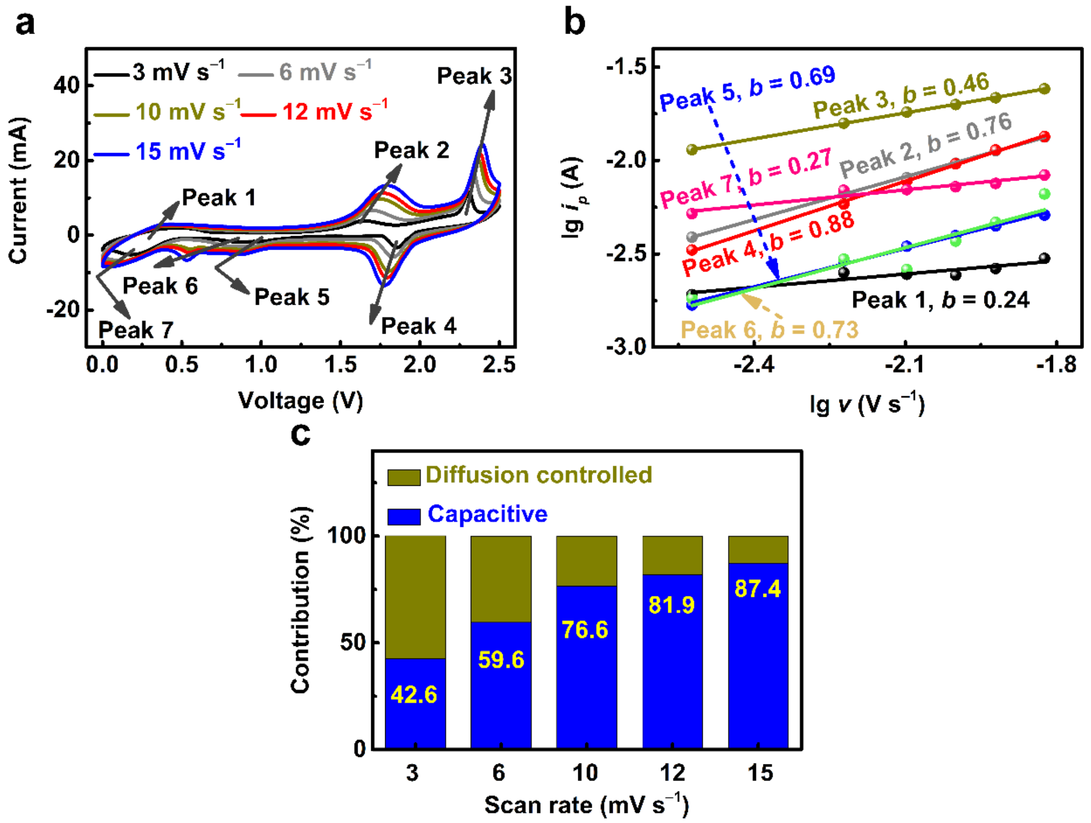

Supplementary Figure 24. Reaction kinetics of the PTCDI||I_2 cascade glass cell. a, CV curves of the PTCDI||I_2 cascade cell in a two-electrode glass cell system (the positive electrode: the I_2 electrode; the negative electrode: the PTCDI electrode; electrolyte: 10 mL saturated mixed KCl/I_2 aqueous solution; 25±1 °C) at various scan rates. b, The corresponding lg i_p *vs*. lg v plots at different redox peaks. c, Contribution ratio of the capacitance- and diffusion-controlled process of the PTCDI||I_2 cascade glass cell at different scan rates.

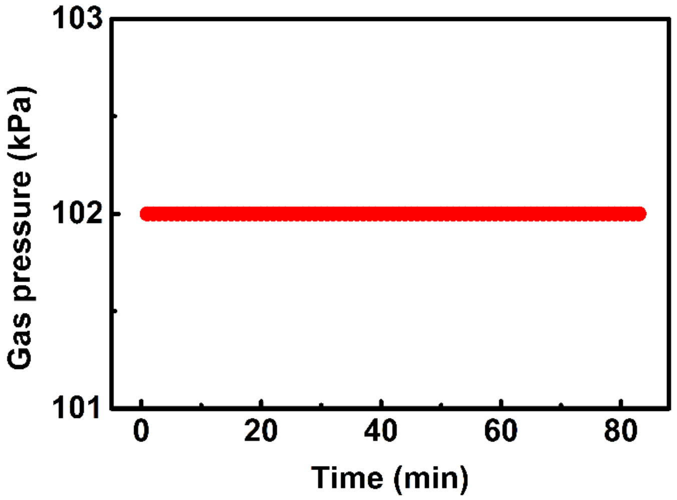

Supplementary Figure 25. In situ pressure test during the CV measurement of the PTCDI||I_2 cascade glass cell. In situ pressure test during the CV measurement of the PTCDI||I_2 cascade cell in a two-electrode glass cell system (the positive electrode: the I_2 electrode; the negative electrode: the PTCDI electrode; electrolyte: 10 mL saturated mixed KCl/I_2 aqueous solution) at 10 mV s^{-1} and 25±1 °C for 10 cycles.

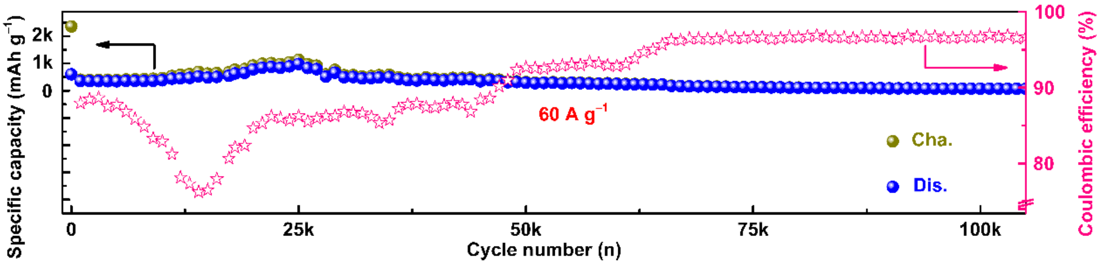

Supplementary Figure 26. Cycling performance of the aqueous PTCDI||I_2 cascade glass cell at 60 A g^{-1}. Cycling performance of the aqueous PTCDI||I_2 cascade cell in a two-electrode glass cell system (the positive electrode: the I_2 electrode; the negative electrode: the PTCDI electrode; electrolyte: 10 mL saturated mixed KCl/I_2 aqueous solution) at 60 A g^{-1} and 25±1 °C.

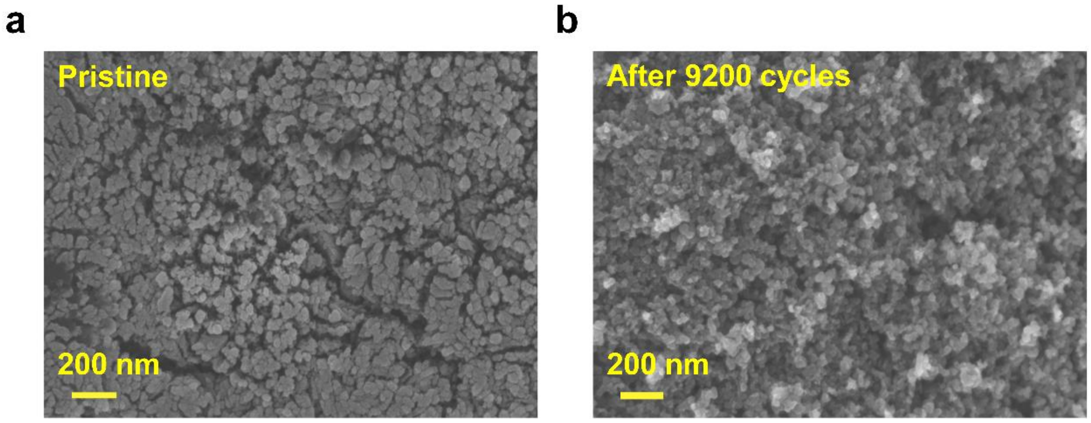

Supplementary Figure 27. Ex situ SEM images of the PTCDI electrode in pristine and after 9000 cycles. Ex situ SEM images of the PTCDI electrode in pristine (uncycled) (a) and (b) after cycling for 9200 cycles at 40 A g^{-1} and 25±1 °C in a two-electrode glass cell system (the positive electrode: the I_2 electrode; the negative electrode: the PTCDI electrode; electrolyte: 10 mL saturated mixed KCl/I_2 aqueous solution).

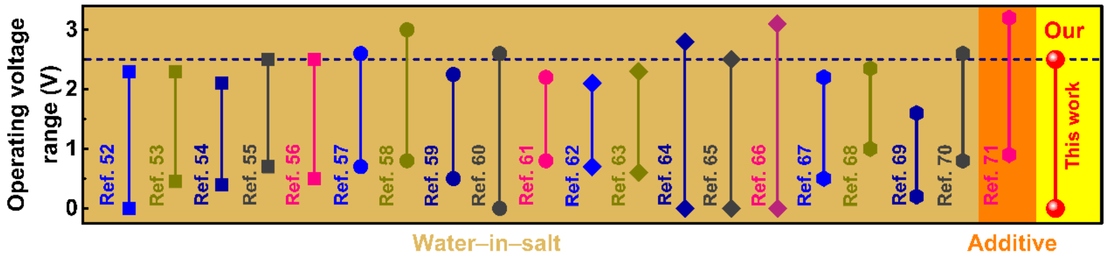

Supplementary Figure 28. Comparison of the operating voltage range between the PTCDI||I_2 cascade cell and the literatures on high-voltage aqueous batteries.

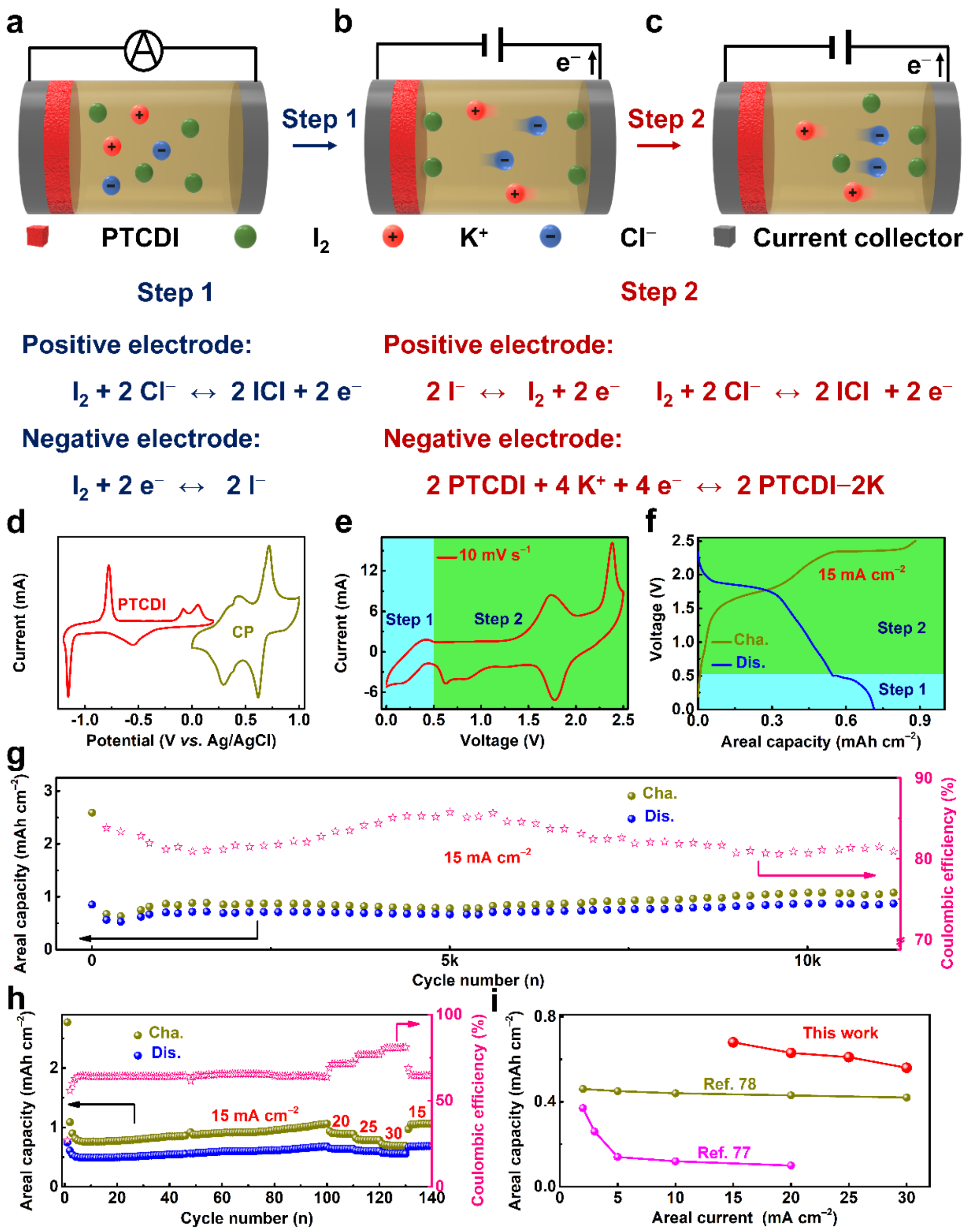

Supplementary Figure 29. Electrochemical performance of the PTCDI||CP cathode-free cascade glass cell. a–c, Schematics of the PTCDI||CP cathode-free cascade glass cell in a saturated mixed KCl/I_2 aqueous electrolyte at the pristine (uncycled), first charge step and second charge step. d, Comparison of CV curves of the PTCDI electrode and the CP electrode in a saturated mixed KCl/I_2 aqueous electrolyte at 10 mV s^{-1} and 25±1 °C. e–h, CV curve, GCD curve collected from the 1000th cycle, cycling performance, and rate capability of the PTCDI||CP cathode-free cascade glass cell at 25±1 °C. i,

Comparison of the rate capability between this work and the literature on cathode-free batteries.

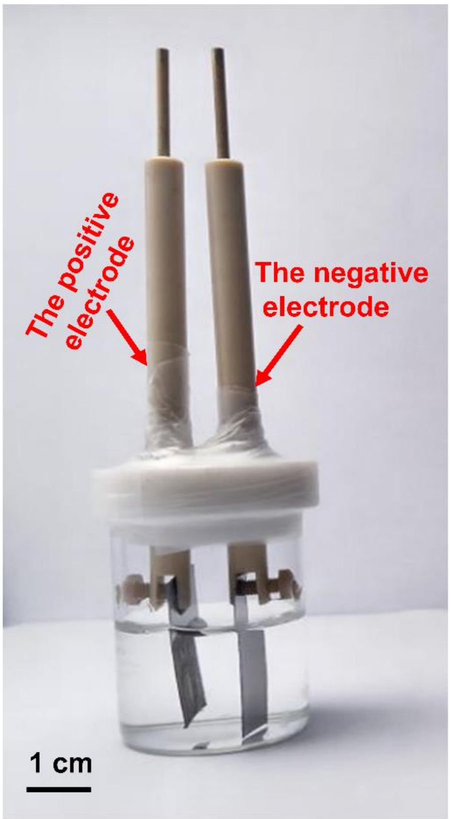

Supplementary Figure 30. Configuration of the assembled two-electrode glass cell. Configuration of the assembled glass cell in an aqueous electrolyte.

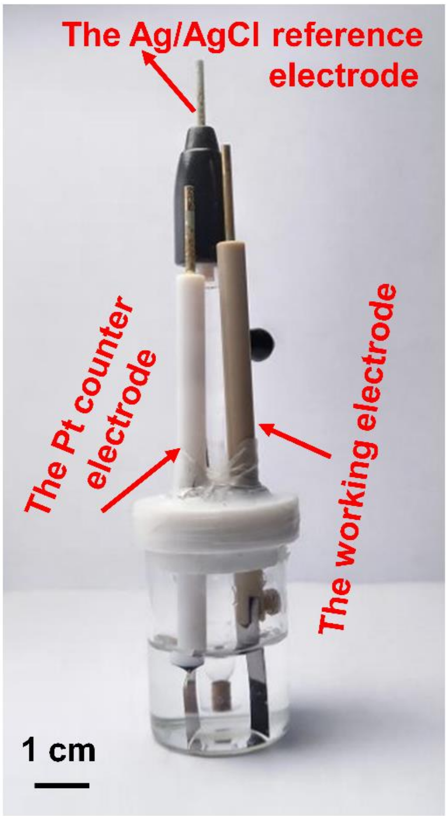

Supplementary Figure 31. Configuration of the assembled three-electrode glass cell. Configuration of the assembled three-electrode glass cell with the platinum (Pt) foil as counter electrode and the standard Ag/AgCl as reference electrode in an aqueous electrolyte.

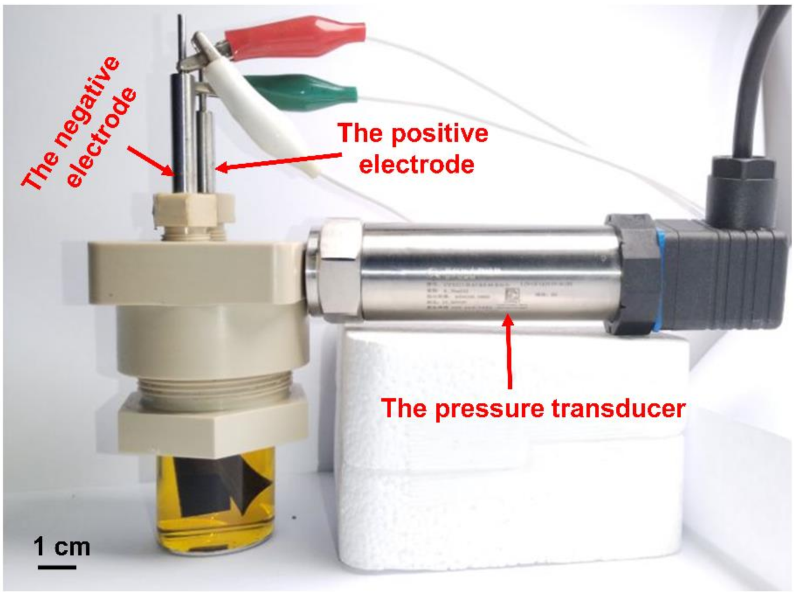

Supplementary Figure 32. The picture of in situ pressure measurements during the CV experiments. The picture of the in-house developed in situ cell used for the pressure measurements during the CV experiments.

Supplementary Table 1. Rietveld refined lattice parameters of the crystalline phase of PTCDI.

Lattice parameters	*a* (nm)	*b* (nm)	*c* (nm)	α (◦)	β (◦)	γ (◦)	V (nm^3)
results	0.508	1.532	1.130	90.000	91.583	90.000	0.879

Supplementary Table 2. The equivalent circuit for the EIS tests of the PTCDI electrode in a saturated mixed KCl/I_2 aqueous electrolyte and the error between the raw and fitted data at different states. Here, the series resistance (R_s) represents the resistance of the electrode, electrolyte and the interface between the electrode and electrolyte. Charge transfer resistance (R_{ct}) is a measure of the difficulty encountered when an electron is shifted from one atom or compound to another atom or compound. The constant phase element (CPE) is a capacitive element with a frequency independent negative phase between a capacitor and a resistor. Warburg resistance (W) models semi-infinite linear diffusion (diffusion in one dimension that is only bounded by a large planar electrode on one side).

The equivalent circuit:

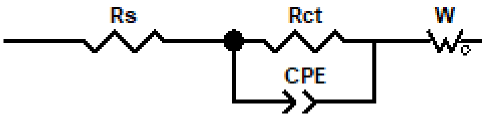

	Fitted value (error) in pristine state	Fitted value (error) after the 3rd cycle	Fitted value (error) after the 4th cycle	Fitted value (error) after the 5th cycle
Series resistance (R_s, Ω)	0.632 (0.002)	0.632 (0.003)	0.633 (0.003)	0.631 (0.004)
Charge transfer resistance (R_{ct}, Ω)	0.159 (0.017)	0.245 (0.066)	0.240 (0.054)	0.229 (0.062)
Constant phase element (CPE)	0.617 (0.031)	0.540 (0.073)	0.644 (0.057)	0.64 (0.035)
Warburg resistance (W, Ω)	0.386 (0.003)	0.436 (0.005)	0.386 (0.004)	0.375 (0.005)

Supplementary Table 3. The equivalent circuit for the EIS tests of the PTCDI||I_2 glass cell in a saturated KCl aqueous electrolyte and the error between the raw and fitted data at different states. Here, the series resistance (R_s) represents the resistance of the electrode, electrolyte and the interface between the electrode and electrolyte. Charge transfer resistance (R_{ct}) is a measure of the difficulty encountered when an electron is shifted from one atom or compound to another atom or compound. The constant phase element (CPE) is a capacitive element with a frequency independent negative phase between a capacitor and a resistor. Warburg resistance (W) models semi-infinite linear diffusion (diffusion in one dimension that is only bounded by a large planar electrode on one side).

The equivalent circuit:

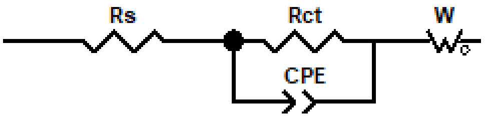

	Fitted value (error) after the 2nd cycle	Fitted value (error) after the 40th cycle	Fitted value (error) after the 90th cycle
Series resistance (R_s, Ω)	1.511 (0.005)	1.501 (0.107)	1.487 (0.112)
Charge transfer resistance (R_{ct}, Ω)	0.341 (0.053)	0.312 (0.041)	0.272 (0.040)
Constant phase element (CPE)	0.660 (0.062)	0.734 (0.066)	0.765 (0.071)
Warburg resistance (W, Ω)	0.335 (0.012)	0.380 (0.004)	0.366 (0.005)

Supplementary Table 4. Comparison of the cycling performance and rate capability between the PTCDI||I_2 glass cell and the previously reported works.

Anode||Cathode	Specific current ($A g^{-1}$)	Specific capacity ($mAh g^{-1}$)	Ref.
G-Zn||ZC-mK_2CO_3@I_2	10.55	150 (after 35k cycles)	1
	0.211	336	
	0.422	328	
	1.055	302	
	2.11	244	
	4.22	200	
	10.761	164	
	21.1	140	
Al||I_2@ZIF-8-C	2	162 (after 150 cycles)	2
	2	240	
	3	190	
	4	150	
	6	110	
	8	90	
Zn||I_2-Nb_2CT_x	6	150 (after 23k cycles)	3
	1	205	
	2	195	
	4	190	
	6	180	
	8	170	
	10	160	
	12	155	
	14	150	
	16	145	
	18	143	
Zn||PAC-I_2	2	420 (after 6k cycles)	4
	0.2	609	
	0.4	589	
	0.8	530	
	1.2	507	
	2	419	
Zn||$Ti_3C_2I_2$	3	125 (after 2.8k cycles)	5
	0.5	198	
	1	180	
	1.5	170	
	2	160	
	2.5	150	
	3	145	
	3.5	140	
	4	135	
	4.5	130	
	5	120	
Zn||$Co[Co_{1/4}Fe_{3/4}(CN)_6]/I_2$	4	166 (after 2k cycles)	6
	0.1	240	
	0.2	228	

	0.5	225	
	1	215	
	2	205	
	4	190	
	6	178	
	20	152	
Zn‖PANI-I_2	1.5	127 (after 700 cycles)	7
	0.3	230	
	0.6	198	
	1.2	171	
	1.8	154	
	2.4	144	
Zn‖I_2-NPC-900	2.11	125 (after 10k cycles)	8
	0.0422	334	
	0.1055	298	
	0.211	261	
	0.422	229	
	1.055	200	
	2.11	178	
	4.22	155	
Zn-BTC‖I_2	1.92	85.1 (after 6k cycles)	9
	0.16	203	
	0.32	163	
	0.64	134	
	1.28	103	
Fe‖I_2/N-HPC	2	195 (after 550 cycles)	10
	0.5	190	
	1	195	
	2	190	
DPPZ‖InHCF, K-ion electrolyte	6	78.3 (after 5k cycles)	11
	1	132	
	2	119	
	3	110	
	4	102	
	5	100	
	6	98	
	7	97	
	8	96	
	9	95	
	10	90	
KTP/C‖K-FeHCF, K-ion electrolyte	5	43 (after 30k cycles)	12
	0.05	80	
	0.1	72	
	0.2	65	
	0.5	60	
	1	55	

	2	45	
	5	44	
PTCDI‖KVO, K-ion electrolyte	1	50 (after 20k cycles)	13
	0.1	95	
	0.2	90	
	0.5	85	
	1	80	
	2	78	
	3	74	
This work	40	289 (after 10k cycles)	
	40	224 (after 40k cycles)	
	40	173 (after 90k cycles)	
	40	321.5	
	70	207	
	100	141	
	130	112	
	160	103	

Supplementary Note 1

XRD and ATR−FTIR measurements for PTCDI powder

The experimental spectrum featuring strong diffraction peaks is identical to the simulated pattern, confirming the high crystallinity and purity of PTCDI (Supplementary Figure 2a). The Rietveld refinement results reveal that the PTCDI features a monoclinic phase with a space group of *P*21/n and cell parameters of a = 0.508 nm, b = 1.532 nm, c = 1.130 nm, α = 90.000°, β = 91.583°, and γ = 90.000° (Supplementary Table 1).[14] The interplanar distance of the PTCDI stack was calculated to be 0.393 nm, which is larger than that of graphite (0.335 nm) and facilitates the diffusion of K−ion (0.138 nm) within the PTCDI crystal (Supplementary Figure 2b).[14, 15] Moreover, the characteristic vibrations of N−H (3153 cm^{-1}), C=O (1686 cm^{-1}), and C−N (1362 and 1276 cm^{-1}) were clearly detected, and the strong absorption peak at 1586 cm^{-1} was assigned to the stretching vibration of C=C bonds in the aromatic ring (Supplementary Figure 2c).[14-18]

Supplementary Note 2

Reaction kinetics of the PTCDI electrode in a saturated KCl aqueous electrolyte

As shown in Supplementary Figure 3a, during the first cathodic scan, one large reduction peak was observed at a more negative potential of −0.76 V than that in subsequent cycles, which was ascribed to the larger polarization needed for the initial electrochemical intercalation of K−ion into the uncoordinated PTCDI electrode.[17] On the first anodic scan, a small shoulder peak located at −0.83 V was observed but disappeared in the following cycles, which may be attributed to some irreversible impurities adsorbed on the electrode.[19] During subsequent cycles, two cathodic peaks at −0.55 V and −1.17 V that originated from the enolization of the carbonyl groups were observed, corresponding to the stepwise intercalation process of K−ion in the PTCDI electrode. Conversely, three anodic peaks appearing at −0.76 V, −0.07 V and 0.06 V, which were derived from the recovery of carbonyl groups, were observed, corresponding to the stepwise deintercalation of K-ion.[16, 20, 21] Moreover, the redox peaks almost overlapped after the first cycle, suggesting the high reversibility of PTCDI

as a K−ion hosting anode. As presented in Supplementary Figure 3b, no redox peaks were observed, verifying that protons had no effect on the charge/discharge process of the PTCDI electrode.

To further elucidate the kinetic behaviours, CV tests were performed at various scan rates from 5 mV s^{-1} to 45 mV s^{-1}, which was a large enough range to evaluate the kinetic behaviour.[5, 22-24] With increasing scan rates, the intensity of the redox peaks increased gradually, and the CV curves retained similar shapes, confirming the excellent high rate tolerance of the PTCDI electrode (Supplementary Figure 3c).[25] To determine whether the relatively broad peak (named peak 4) within −0.8 V to −0.3 V consists of one peak or not, the PTCDI electrode was tested in a three-electrode system at an ultralow scan rate of 0.2 mV s^{-1}. As presented in Supplementary Figure 3d, it is clear that peak 4 consists of one peak. The broadening of the peaks at high scan rates should be mainly attributed to the increased polarization.[5, 15, 22-24]

In principle, the relationship between the peak current (i_p) and scan rate (v) can be expressed by the power law:[26-28]

$$i = av^b \tag{1}$$

where i and v represent the generated current and applied scan rate, respectively, and a and b are constants. In detail, b ~0.5 indicates a diffusion-controlled process, while b ~1 indicates a capacitance-dominated process.[26-28] By plotting lg i_p *vs*. lg v (Supplementary Figure 3e), the b values of the five marked redox peaks are calculated to be 0.60 (peak 1), 0.79 (peak 2), 0.80 (peak 3), 0.80 (peak 4), and 0.54 (peak 5), manifesting the combination of diffusion-controlled and capacitance-dominated behaviours of the whole redox process.[29]

For further determination of the capacitive contribution, the current density (i) at a fixed potential can be divided into the capacitance-dominated contribution (k_1v) and the diffusion effect ($k_2v^{1/2}$), and the relationship can be expressed as follows:[26-28]

$$i = k_1v + k_2v^{1/2} \tag{2}$$

As a typical example, at a scan rate of 15 mV s^{-1}, the capacitance-controlled contribution approached 69.0%. Moreover, the proportion of capacitive-domination

increased with increasing scan rate and reached 83.9% at 45 mV s^{-1} (Supplementary Figure 3f). The high capacitive contribution of the PTCD electrode is a common phenomenon and has been widely reported in previous works.[14, 16, 17, 20, 30-33] The high capacitive contribution means that the kinetic behaviour of the electrode is mainly dominated by the capacitive effect, and the transport of K-ion is not the rate-limiting factor.[32] In other words, the transport of K–ion is fast enough under high operational current densities. In addition, the capacitive effect renders more charge transfer than volume lattice diffusion and thus can help to retain the capacity at high operational current densities.[34, 35]

Supplementary Note 3

Electrochemical performance of the PTCDI electrode

As displayed in Supplementary Figure 4, after the first few cycles, a capacity of 136 mAh g^{-1} was delivered, corresponding to the two–electron redox mechanism per PTCDI molecule (137 mAh g^{-1} in theory).[14, 17, 20]

Supplementary Note 4

The reaction mechanism of the PTCDI electrode

By combining the aforementioned CV results and actual capacity delivered, the redox mechanism of the PTCDI electrode can be expressed as a stepwise enolization reaction in terms of reversible stepwise intercalation of K-ion (Supplementary Figure 5).[14, 17, 20]

Supplementary Note 5

Reaction kinetics of the PTCDI electrode in a saturated mixed KCl/I_2 aqueous electrolyte

As presented in Supplementary Figure 6a, the CV curves of the PTCDI electrode in the saturated mixed KCl/I_2 aqueous electrolyte were identical to those in the pure saturated KCl electrolyte (Supplementary Figure 3a), demonstrating the intrinsic

characteristics of inertness to various iodine anionic species of the PTCDI electrode. Since the voltage at the beginning of the test was 0.16 V, the curves in the vicinity of 0.2 V between the 1st scan cycle and the sequential scan cycles were nonclosed. To determine the variation in the ohmic resistance of the PTCDI electrode after different scan cycles, corresponding electrochemical impedance spectroscopy (EIS) measurements were performed. As shown in Supplementary Figure 6b, the intersection of the EIS curve with the horizontal axis after different scan cycles effectively overlapped, illustrating that the series resistance of the PTCDI electrode remained stable without significant variation and consolidating that the PTCDI electrode can work stably in the mixed electrolyte.[6, 36-38]

Supplementary Note 6

BET and TGA measurements of AC and I_2@AC powder

As shown in Supplementary Figure 7a, the specific surface area of AC decreased from 2087.5 m^2 g^{-1} to 924.5 m^2 g^{-1} after adsorbing I_2, verifying the successful preparation of the I_2@AC material. As presented in Supplementary Figure 7b, the second stage corresponds to the mass loss of I_2, and the content of I_2 in the I_2@AC was determined to be 47.2%.

Supplementary Note 7

Reaction kinetics of the PTCDI||I_2 glass cell in a saturated KCl aqueous electrolyte

As presented in Supplementary Figure 9a, there are two cathodic peaks below 1.2 V after the 2nd scan, but the lower potential position of the cathodic peaks overlapped with the 40th scan curve. For clarity, the cathodic peaks below 1.2 V after the 2nd scan was enlarged (Supplementary Figure 9b). It is clear that there are two cathodic peaks below 1.2 V after the 2nd scan. To investigate the phenomenon of the shift in peak potential of the full cell, electrochemical impedance spectroscopy (EIS) measurements were performed after the 2nd, 40th and 90th scans at 10 mV s^{-1}. As shown in Supplementary Figure 9c, the EIS curves after different scan cycles presented similar

shapes and the positions shifted slightly to the left with the increased scan cycles. Notably, the intersection of the EIS curve with the horizontal axis presents the series resistance of the cell.[6, 36-38] The smaller the intercept was, the smaller the series resistance.[6, 36-38] To make it clearer, the inset shows the enlarged zone of the curves at high frequency. It is obvious that the ohmic resistance of the cell decreased with increasing scan cycles.

Various scan rates from 2 mV s^{-1} to 30 mV s^{-1} for the PTCDI||I_2 glass cell in saturated KCl electrolyte were tested, and the width of the scan rates was large enough to evaluate the kinetic behaviour.[5, 22-24] With increasing scan rate, the shape of the CV curves was well maintained (Supplementary Figure 9d). To determine whether the relatively broad peak (named peak 3) within 1.8 V to 2.1 V consists of one peak or not, the full cell was tested at a small scan rate of 1 mV s^{-1}. As presented in Supplementary Figure 9e, peak 3 consists of one peak. The broadening of the peaks at high scan rates should be mainly attributed to the increased polarization.[5, 15, 22-24] Moreover, the b values of the redox peaks were calculated to be 0.64 (peak 1), 0.52 (peak 2), 0.78 (peak 3), 0.64 (peak 4), and 0.68 (peak 5) according to equation (1), suggesting the combined mechanism of the whole redox process (Supplementary Figure 9f). As a typical example, at a scan rate of 10 mV s^{-1}, the capacitance-controlled contribution approached 53.3%. Moreover, it increased with increasing scan rate and reached 72.1% at 30 mV s^{-1} (Supplementary Figure 9g), implying the outstanding rate performance of the full cell. [34, 35]

Supplementary Note 8

GCD curve of the PTCDI||I_2 glass cell within 0.3-2.4 V

To ensure that all redox peaks can be completely displayed, the voltage range of the PTCDI||I_2 glass cell was set to 0.3-2.4 V. As shown in Supplementary Figure 10, a discharge capacity of 300 mAh g^{-1} was delivered, which still far exceeded that of all reported aqueous iodine-cathode batteries at such a high current density.[1, 3-9] An additional discharge capacity of 24 mAh g^{-1} was delivered when the cut-off voltage of

the full cell was set to 0, which only accounted for 7.4% of the total capacity (324 mAh g^{-1} in Figure 2b). Therefore, the cut-off voltage of the full cell was not the main reason for the high capacity delivered. Instead, a slightly higher capacity output was obtained if the cut-off voltage was set to 0, and the same method has also been reported in previous works.[15, 20, 33]

Supplementary Note 9

Comparison of the maximum discharge voltage plateau between the PTCDI||I_2 glass cell and reported works

The maximum discharge voltage plateau of the PTCDI||I_2 glass cell is higher than that of all reported aqueous rechargeable Zn||I_2,[1, 3-9] Fe||I_2[10], Al||I_2,[2] H_2||I_2,[39] and most aqueous rechargeable K-ion full battery systems (ARKFBs),[11, 12, 40] making it promising for high-energy output (Supplementary Figure 11).

Supplementary Note 10

GCD curve of the PTCDI||I_2 glass cell at 1 A g^{-1}

As presented in Supplementary Figure 12, the performance of the PTCDI||I_2 glass cell at a low specific current (1 A g^{-1}) was investigated. The cell was unable to be charged to 2.4 V but fluctuated in the vicinity of 1.8 V. This result was mainly attributed to some irreversible side reactions induced under a low specific current and the limited intrinsic conductivity of organic electrode materials.[15, 20, 32, 33, 41] Thus, a high specific current was needed to ensure that the required voltage was reached and that some irreversible side reactions were avoided. A similar phenomenon has also been reported in other works.[15, 20, 32]

Supplementary Note 11

Comparison of the self-discharge rate between the PTCDI||I_2 glass cell and Zn||I_2 glass cell

The self-discharge rate of the PTCDI||I_2 glass cell was evaluated after charging to 2.4 V at 40 A g^{-1}. To obtain a better understanding of the self-discharge rate of this work, a Zn||I_2 glass cell was constructed in a mixed 1 M KCl + 1 M $ZnCl_2$ aqueous electrolyte and evaluated after charging to 1.8 V at 40 A g^{-1} as a control group.[5, 42] As shown in Supplementary Figure 14, a voltage drop of 0.4 V for the PTCDI||I_2 glass cell required 78.2 s, while the time for the same voltage drop for the Zn||I_2 glass cell was 27.6 s. That is, the PTCDI||I_2 glass cell had a much lower self-discharge rate than that of the conventional Zn||I_2 glass cell.

Self-discharge behavior is a common phenomenon in aqueous iodine cathode batteries, and is ascribed to the dissolubility of iodine and iodine species in aqueous environments.[3-5, 42-44] In other words, regardless of which kind of iodine electrode material is utilized as the cathode, iodine species are inevitably released into the aqueous electrolyte and trigger a series of complexation reactions, thus leading to self-discharge behaviour.[3-5, 42-44] For conventional aqueous metal||I_2 battery systems, iodine anionic species diffuse into the vicinity of the metal anode and cause the formation of electrochemically inactive complexes, leading to the irreversible loss of iodine elements and aggravating the self-discharge behaviour.[3-5, 42-44]

In contrast, PTCDI featuring intrinsic inertness to various iodine anionic species was adopted as the negative electrode in this work. They were unable to react with the PTCDI electrode and form electrochemically inactive complexes. Hence, the PTCDI||I_2 glass cell in this work had a lower self-discharge rate than that of the conventional Zn||I_2 glass cell. Although coupled with the simplest iodine cathode material (I_2@AC) synthesized through a facile physical adsorption method, the aqueous PTCDI||I_2 glass cell still exhibited an improved electrochemical performance.

Supplementary Note 12

The specific energy and power performances of the PTCDI||I_2 glass cell compared to the previously reported works.

As presented in Supplementary Figure 15 (the specific energy and specific power were both calculated based on the mass of the active material in the positive electrode), the full cell exhibited a specific energy of 434 Wh kg^{-1} at a specific power of 50420 W kg^{-1}. Moreover, a specific energy of 86 Wh kg^{-1} was maintained at the peak specific power of 155072 W kg^{-1}. Furthermore, the specific energy and specific power of the full cell are higher than that reported for aqueous batteries with I_2-based cathodes[1, 3, 6, 10, 44-48] and most ARKFBs.[11, 49]

Supplementary Note 13

CV curve of the I_2 electrode in a three-electrode glass cell system

As shown in Supplementary Figure 16, the CV of the I_2 electrode in a saturated KCl electrolyte was tested in a three-electrode glass cell system. Only a pair of redox peaks corresponding to I^-/I^0 was observed, and no other redox peaks corresponding to I^-/I_3^- were detected, confirming a direct conversion between I^- and I^0.[4, 5, 50] These results are consistent with the DFT result in Figure 1a.[3-6, 51]

Supplementary Note 14

LSV curve of the saturated mixed KCl/I_2 aqueous electrolyte

As shown in Supplementary Figure 17, the slight bulge within the potential range of 0.6 V to 0.9 V corresponds to the oxidation reaction of I^0/I^+ in the mixed electrolyte,[4, 5] which is an indispensable part of the $I^-/I^0||I^0/I^+$ redox system. Hence, the electrochemical potential window of the mixed electrolyte was determined to be 2.60 V, which is large enough to guarantee the stable work of the cascade cell.

Supplementary Note 15

Reaction kinetics of the PTCDI||I_2 cascade glass cell in a saturated mixed KCl/I_2 aqueous electrolyte

Various scan rates from 3 mV s^{-1} to 15 mV s^{-1} for the PTCDI||I_2 cascade cell were used to evaluate the kinetic behaviour.[23, 24] As presented in Supplementary Figure 24a,

the CV curves displayed similar shapes, confirming the excellent high rate tolerance of the cascade cell.[25] According to equation (1), the *b* values of the seven marked redox peaks were calculated to be 0.24 (peak 1), 0.76 (peak 2), 0.46 (peak 3), 0.88 (peak 4), 0.69 (peak 5), 0.73 (peak 6), and 0.27 (peak 7), indicating the combination of diffusion-controlled and capacitance-dominated behaviours of the whole redox process (Supplementary Figure 24b).[29]

Supplementary Note 16

Comparison of the operating voltage range between the PTCDI||I_2 cascade glass cell and the reported works

As shown in Supplementary Figure 28, compared with well-known strategies (e.g., water-in-salt, liquid polymer additive)[52-71] to broaden the voltage window in aqueous electrolytes, the high voltage of the PTCDI||I_2 cascade cell was achieved by just utilizing the saturated mixed KCl/I_2 aqueous electrolyte without a water-in-salt strategy or polymers.

Supplementary Note 17

Electrochemical performance of the PTCDI||CP cathode-free cascade glass cell

As illustrated in Supplementary Figure 29d, the carbon paper (CP) cathode (functioning as a current collector) and PTCDI anode can work steadily in the mixed electrolyte. It is noted that the discharge areal capacity and areal current density values of the PTCDI||CP cathode-free cascade glass cell were calculated based on the total area (~2 cm^2) of the both sides of the positive electrode. As expected, the typical CV curve of the PTCDI||CP cathode−free cascade cell is in accord with that of the PTCDI||I_2 cascade cell (Supplementary Figure 29e). Correspondingly, two distinct procedures (marked step 1 and step 2 separately) were clearly observed in the GCD curve (Supplementary Figure 29f). As shown in Supplementary Figure 29g, a discharge areal capacity of 0.87 mAh cm^{-2} was delivered at 15 mA cm^{-2} after 11200 cycles, exhibiting stable cycling performance. The fluctuant CE values were attributed to the cathode

reactions of $I^-/I^0/I^+$ occurring at the interface between the CP current collector and electrolyte for the two steps of this cascade cell.[72-76] Moreover, discharge areal capacities of 0.67, 0.66, 0.61 and 0.57 mAh cm^{-2} were achieved at 15, 20, 25 and 30 mA cm^{-2}, respectively, indicating a good rate capability (Supplementary Figure 29h). Impressively, the rate capability of the PTCDI||CP cathode-free cascade cell displayed prominent superiority when compared with other reported cathode-free batteries (Supplementary Figure 29i).[77, 78]

Supplementary References

1. Chen, C. et al. High-energy density aqueous zinc-iodine batteries with ultra-long cycle life enabled by the ZnI_2 additive. *ACS Sustain. Chem. Eng.* **9**, 13268-13276 (2021).
2. Yang, S. et al. High-rate aqueous aluminum-ton batteries enabled by confined iodine conversion chemistry. *Small Methods* **5**, 2100611 (2021).
3. Li, X. et al. Enhanced redox kinetics and duration of aqueous I_2/I^- conversion chemistry by mxene confinement. *Adv. Mater.* **33**, 2006897 (2021).
4. Zou, Y. et al. A four-electron Zn-I_2 aqueous battery enabled by reversible $I^-/I_2/I^+$ conversion. *Nat. Commun.* **12**, 170 (2021).
5. Li, X. et al. Activating the I^0/I^+ redox couple in an aqueous I_2-Zn battery to achieve a high voltage plateau. *Energy Environ. Sci.* **14**, 407-413 (2021).
6. Ma, L. et al. Electrocatalytic iodine reduction reaction enabled by aqueous zinc-iodine battery with improved power and energy densities. *Angew. Chem. Int. Ed.* **60**, 3791-3798 (2021).
7. Zeng, X. et al. Anchoring polyiodide to conductive polymers as cathode for highperformance aqueous zinc-iodine batteries. *ACS Sustain. Chem. Eng.* **8**, 14280-14285 (2020).
8. Yu, D., Kumar, A., Tuan Anh, N., Nazir, M. T. & Yasin, G. High-voltage and ultrastable aqueous zinc-iodine battery enabled by N-doped carbon materials: revealing the contributions of nitrogen configurations. *ACS Sustain. Chem. Eng.* **8**, 13769-13776 (2020).
9. Yang, H. et al. A metal-organic framework as a multifunctional ionic sieve membrane for long-life aqueous zinc-iodide batteries. *Adv. Mater.* **32**, 2004240 (2020).
10. Bai, C., Jin, H., Gong, Z., Liu, X. & Yuan, Z. A high-power aqueous rechargeable Fe-I_2 battery. *Energy Stor. Mater.* **28**, 247-254 (2020).
11. Qiao, J. et al. Long-life aqueous H^+/K^+ dual-cation batteries based on dipyridophenazine//hexacyanoferrate electrodes. *ACS Appl. Energy Mater.* **4**,

4903-4909 (2021).

12. Li, Y. et al. An ultra-long life aqueous full K-ion battery. *J. Mater. Chem. A* **9**, 2822-2829 (2021).

13. Liang, G. et al. Reconstructing vanadium oxide with anisotropic pathways for a durable and fast aqueous K-ion battery. *ACS Nano* **15**, 17717-17728 (2021).

14. Bai, Y. et al. Perylenetetracarboxylic diimide as a high-rate anode for potassium-ion batteries. *J. Mater. Chem. A* **7**, 24454-24461 (2019).

15. Han, C., Li, H., Li, Y., Zhu, J. & Zhi, C. Proton-assisted calcium-ion storage in aromatic organic molecular crystal with coplanar stacked structure. *Nat. Commun.* **12**, (2021).

16. Wu, D. et al. An acid-pasting approach towards perylenetetracarboxylic diimide based lithium/sodium ion battery cathodes with high rate performances. *J. Colloid Interface Sci.* **538**, 597-604 (2019).

17. Deng, W., Shen, Y., Qian, J., Cao, Y. & Yang, H. A perylene diimide crystal with high capacity and stable cyclability for Na-ion batteries. *ACS Appl. Mater. Interfaces* **7**, 21095-21099 (2015).

18. Lei, X., Zheng, Y., Zhang, F., Wang, Y. & Tang, Y. Highly stable magnesium-ion-based dual-ion batteries based on insoluble small-molecule organic anode material. *Energy Stor. Mater.* **30**, 34-41 (2020).

19. Zhang, Z. et al. Aqueous rechargeable dual-ion battery based on fluoride ion and sodium ion electrochemistry. *J. Mater. Chem. A* **6**, 8244-8250 (2018).

20. Jiang, L. et al. Building aqueous K-ion batteries for energy storage. *Nat. Energy* **4**, 495-503 (2019).

21. Xiong, M., Tang, W., Cao, B., Yang, C. & Fan, C. A small-molecule organic cathode with fast charge-discharge capability for K-ion batteries. *J. Mater. Chem. A* **7**, 20127-20131 (2019).

22. Jin, X. et al. A flexible aqueous zinc-iodine microbattery with unprecedented energy density. *Adv. Mater.* **34**, 2109450 (2022).

23. Cheng, Z. et al. Achieving long cycle life for all-solid-state rechargeable Li-I_2

battery by a confined dissolution strategy. *Nat. Commun.* **13**, 125 (2022).

24. Li, X. et al. Intrinsic voltage plateau of a $Nb_{(2)}CT_x$ MXene cathode in an aqueous electrolyte induced by high-voltage scanning. *Joule* **5**, 2993-3005 (2021).
25. Sun, B. et al. A passionfruit-like carbon-confined Cu_2ZnSnS_4 anode for ultralong-life sodium storage. *Adv. Energy Mater.* **11**, 2100082 (2021).
26. Chao, H. et al. Boosting the pseudocapacitive and high mass-loaded lithium/sodium storage through bonding polyoxometalate nanoparticles on MXene nanosheets. *Adv. Funct. Mater.* **31**, 2007636 (2021).
27. Yue, F. et al. An ultralow temperature aqueous battery with proton chemistry. *Angew. Chem. Int. Ed.* **60**, 13882-13886 (2021).
28. Chen, H. et al. Interlayer modification of pseudocapacitive vanadium oxide and $Zn(H_2O)_{(n)}^{(2+)}$ migration regulation for ultrahigh rate and durable aqueous zinc-ion batteries. *Adv. Sci.* **8**, 2004924 (2021).
29. Song, Y. et al. Ammonium-ion storage using electrodeposited manganese oxides. *Angew. Chem. Int. Ed.* **60**, 5718-5722 (2021).
30. Yang, L. et al. Flexible and additive-free organic electrodes for aqueous sodium ion batteries. *J. Mater. Chem. A* **8**, 22791-22801 (2020).
31. Liu, N. et al. Building high rate capability and ultrastable dendrite-free organic anode for rechargeable aqueous zinc batteries. *Adv. Sci.* **7**, 2000146 (2020).
32. Liebl, S. et al. Perylenetetracarboxylic diimide as diffusion-less electrode material for high-rate organic Na-ion batteries. *Chem. Eur. J.* **26**, 17559-17566 (2020).
33. Ge, J., Fan, L., Rao, A. M., Zhou, J. & Lu, B. Surface-substituted prussian blue analogue cathode for sustainable potassium-ion batteries. *Nat. Sustain.* **5**, 225-234 (2022).
34. Chao, D. & Fan, H. J. Intercalation pseudocapacitive behavior powers aqueous batteries. *Chem* **5**, 1359-1361 (2019).
35. Chao, D. et al. Array of nanosheets render ultrafast and high-capacity na-ion storage by tunable pseudocapacitance. *Nat. Commun.* **7**, 12122 (2016).

36. Geng, H. et al. Electronic structure regulation of layered vanadium oxide via interlayer doping strategy toward superior high-rate and low-temperature zinc-ion batteries. *Adv. Funct. Mater.* **30**, 1907684 (2020).
37. Yang, D. et al. A manganese phosphate cathode for long-life aqueous energy storage. *Adv. Funct. Mater.* **31**, 2100477 (2021).
38. Ma, G. et al. Li-ion storage properties of two-dimensional titanium-carbide synthesized via fast one-pot method in air atmosphere. *Nat. Commun.* **12**, 5085 (2021).
39. Zhu, Z., Meng, Y., Cui, Y. & Chen, W. An ultrastable aqueous iodine-hydrogen gas battery. *Adv. Funct. Mater.* **31**, 2101024 (2021).
40. Wang, M., Wang, H., Zhang, H. & Li, X. Aqueous K-ion battery incorporating environment-friendly organic compound and berlin green. *J. Energy Chem.* **48**, 14-20 (2020).
41. Li, M. et al. Halogenated Ti_3C_2 MXenes with electrochemically active terminals for high-performance zinc ion batteries. *ACS Nano* **15**, 1077-1085 (2021).
42. Ma, J., Liu, M., He, Y. & Zhang, J. Iodine redox chemistry in rechargeable batteries. *Angew. Chem. Int. Ed.* **60**, 12636-12647 (2021).
43. Zhao, Q., Lu, Y., Zhu, Z., Tao, Z. & Chen, J. Rechargeable lithium-iodine batteries with iodine/nanoporous carbon cathode. *Nano Lett.* **15**, 5982-5987 (2015).
44. Pan, H. et al. Controlling solid-liquid conversion reactions for a highly reversible aqueous zinc-iodine battery. *ACS Energy Lett.* **2**, 2674-2680 (2017).
45. Li, W., Wang, K. & Jiang, K. A high energy efficiency and long life aqueous Zn-I_2 battery. *J. Mater. Chem. A* **8**, 3785-3794 (2020).
46. Li, H., Li, M., Zhou, X. & Li, T. A novel rechargeable iodide ion battery with zinc and copper anodes. *J. Power Sources* **449**, 227511 (2020).
47. Lu, K. et al. Sulfur and nitrogen enriched graphene foam scaffolds for aqueous rechargeable zinc-iodine battery. *Electrochim. Acta* **296**, 755-761 (2019).

48. Bai, C. et al. A sustainable aqueous Zn-I_2 battery. *Nano Res.* **11**, 3548-3554 (2018).

49. Wessells, C. D., Peddada, S. V., Huggins, R. A. & Cui, Y. Nickel hexacyanoferrate nanoparticle electrodes for aqueous sodium and potassium ion batteries. *Nano Lett.* **11**, 5421-5425 (2011).

50. Li, X. et al. Two-electron redox chemistry enabled high-performance iodide-ion conversion battery. *Angew. Chem. Int. Ed.* **61**, e202113576 (2022).

51. Yang, Y., Liang, S., Lu, B. & Zhou, J. Eutectic electrolyte based on N-methylacetamide for highly reversible zinc-iodine battery. *Energy Environ. Sci.* **15**, 1192-1200 (2022).

52. Zhang, C. et al. A $ZnCl_2$ water-in-salt electrolyte for a reversible Zn metal anode. *Chem. Commun.* **54**, 14097-14099 (2018).

53. Suo, L. et al. "Water-in-salt" electrolyte enables high-voltage aqueous lithium-ion chemistries. *Science* **350**, 938-943 (2015).

54. Yue, J. et al. Aqueous interphase formed by CO_2 brings electrolytes back to salt-in-water regime. *Nat. Chem.* **13**, 1061-1069 (2021).

55. Lukatskaya, M. R. et al. Concentrated mixed cation acetate "water-in-salt" solutions as green and low-cost high voltage electrolytes for aqueous batteries. *Energy Environ. Sci.* **11**, 2876-2883 (2018).

56. Hou, X. et al. Simultaneous formation of interphases on both positive and negative electrodes in high-voltage aqueous lithium-ion batteries. *Small* **18**, 2104986 (2021).

57. Nian, Q. et al. An overcrowded water-ion solvation structure for a robust anode interphase in aqueous lithium-ion batteries. *ACS Appl. Mater. Interfaces* **13**, 51048-51056 (2021).

58. Zhou, A. et al. TiO_2 (B) anode for high-voltage aqueous Li-ion batteries. *Energy Stor. Mater.* **42**, 438-444 (2021).

59. Shi, H.-Y. et al. Accessing the 2 V V^{v}/V^{iv} redox process of vanadyl phosphate cathode for aqueous batteries. *J. Power Sources* **507**, 230270 (2021).

60. Hou, X. et al. Stabilizing the solid-electrolyte interphase with polyacrylamide for high-voltage aqueous lithium-ion batteries. *Angew. Chem. Int. Ed.* **60**, 22812-22817 (2021).

61. Pan, W. et al. High-energy SWCNT cathode for aqueous Al-ion battery boosted by multi-ion intercalation chemistry. *Adv. Energy Mater.* **11**, 2101514 (2021).

62. Pan, W. et al. High-performance MnO_2/Al battery with in situ electrochemically reformed Al_xMnO_2 nanosphere cathode. *Small Methods* **5**, 2100491 (2021).

63. Wang, X. et al. High-voltage aqueous planar symmetric sodium ion micro-batteries with superior performance at low-temperature of −40°C ? *Nano Energy* **82**, 105688 (2021).

64. Li, Y. et al. A superconcentrated water-in-salt hydrogel electrolyte for high-voltage aqueous potassium-ion batteries. *Chemelectrochem* **8**, 1451-1454 (2021).

65. Hou, X. et al. TiO_2@$LiTi_2(PO_4)_{(3)}$ enabling fast and stable lithium storage for high voltage aqueous lithium-ion batteries. *J. Power Sources* **484**, 229255 (2021).

66. Li, T., Li, M., Li, H. & Zhao, H. High-voltage and long-lasting aqueous chlorine-ion battery by virtue of "water-in-salt" electrolyte. *Iscience* **24**, 101976 (2021).

67. Reber, D., Grissa, R., Becker, M., Kuhnel, R.-S. & Battaglia, C. Anion selection criteria for water-in-salt electrolytes. *Adv. Energy Mater.* **11**, 2002913 (2021).

68. Zhao, J. et al. High-voltage Zn/$LiMn_{0.8}Fe_{0.2}PO_4$ aqueous rechargeable battery by virtue of "water-in-salt" electrolyte. *Electrochem. Commun.* **69**, 6-10 (2016).

69. Hu, P. et al. Zn/V_2O_5 aqueous hybrid-ion battery with high voltage platform and long cycle life. *ACS Appl. Mater. Interfaces* **9**, 42717-42722 (2017).

70. Yang, C. et al. Flexible aqueous Li-ion battery with high energy and power densities. *Adv. Mater.* **29**, 1701972 (2017).

71. Xie, J., Liang, Z. & Lu, Y.-C. Molecular crowding electrolytes for high-voltage aqueous batteries. *Nat. Mater.* **19**, 1006-1011 (2020).

72. Gao, L. et al. A high-performance aqueous zinc-bromine static battery. *Iscience* **23**, 101348 (2020).

73. Chun, S.-E. et al. Design of aqueous redox-enhanced electrochemical capacitors with high specific energies and slow self-discharge. *Nat. Commun.* **6**, 7818 (2015).

74. Lee, J.-H. et al. High-energy efficiency membraneless flowless Zn-Br battery: utilizing the electrochemical-chemical growth of polybromides. *Adv. Mater.* **31**, 1904524 (2019).

75. Soloveichik, G. L. Flow batteries: Current status and trends. *Chem. Rev.* **115**, 11533-11558 (2015).

76. Xie, C., Duan, Y., Xu, W., Zhang, H. & Li, X. A low-cost neutral zinc-iron flow battery with high energy density for stationary energy storage. *Angew. Chem. Int. Ed.* **56**, 14953-14957 (2017).

77. Chai, S., Zhu, J., Jiang, J. & Li, C. M. Elevating kinetics of passivated fe anodes with NH_4Cl regulator: toward low-cost, long-cyclic and green cathode-free Fe-ion aqueous batteries. *Nano Res.* **15**, 3187-3194 (2021).

78. Liu, Z., Yang, Y., Liang, S., Lu, B. & Zhou, J. pH-buffer contained electrolyte for self-adjusted cathode-free Zn-MnO_2 batteries with coexistence of dual mechanisms. *Small Structures* **2**, 2100119 (2021).
